# Novel Role of miR-18a-5p and Galanin in Rat Lung Ischemia Reperfusion-Mediated Response

**DOI:** 10.1155/2021/6621921

**Published:** 2021-08-14

**Authors:** Kun Xiao, Lei Song, Ye Hu, Wanxue He, Fei Hou, Peng Yan, Jianqiao Xu, Kaifei Wang, Yi Tao, Dan Li, Lixin Xie

**Affiliations:** ^1^Center of Pulmonary & Critical Care Medicine, Chinese People's Liberation Army (PLA) General Hospital, Beijing, China; ^2^Medical School of Chinese People's Liberation Army (PLA), Chinese PLA General Hospital, Beijing, China; ^3^Department of Respiratory Medicine, The First Hospital of Jilin University, Changchun 130061, China

## Abstract

Lung ischemia reperfusion (IR) is known to occur after lung transplantation or cardiac bypass. IR leads to tissue inflammation and damage and is also associated with increased morbidity and mortality. Various receptors are known to partake in activation of the innate immune system, but the downstream mechanism of tissue damage and inflammation is yet unknown. MicroRNAs (miRNAs) are in the forefront in regulating ischemia reperfusion injury and are involved in inflammatory response. Here, we have identified by high-throughput approach and evaluated a distinct set of miRNAs that may play a role in response to IR in rat lung tissue. The top three differentially expressed miRNAs were validated through quantitative PCRs in the IR rat lung model and an *in vitro* model of IR of hypoxia and reoxygenation exposed type II alveolar cells. Among the miRNAs, miR-18a-5p showed consistent downregulation in both the model systems on IR. Cellular and molecular analysis brought to light a crucial role of this miRNA in ischemia reperfusion. miR-18a-5p plays a role in IR-mediated apoptosis and ROS production and regulates the expression of neuropeptide Galanin. It also influences the nuclear localization of transcription factor: nuclear factor-erythroid 2-related factor (Nrf2) which in turn may regulate the expression of the miR-18a gene. Thus, we have not only established a rat model for lung IR and enumerated the important miRNAs involved in IR but have also extensively characterized the role of miR-18a-5p. This study will have important clinical and therapeutic implications for and during transplantation procedures.

## 1. Introduction

Patients with end-stage lung diseases or pulmonary disorders receive lung transplantation as a standard mode of treatment modality. Lung transplantation, even though well established, has a low success rate compared to other solid organ transplants as it poses challenges in terms of survival of donor transplant in the recipient body [1]. This is mostly due to a clinical condition called primary graft dysfunction (PGD), which results from mild or acute lung injury posttransplantation [2]. The major cause of primary graft dysfunction in the recipient body is due to the injury during ischemia reperfusion and the clinical symptoms including pulmonary edema and alveolar damage [3]. It is reviewed that lung ischemia reperfusion-related injury plays a major role in determining the success rate of lung transplantation procedures contributed by increased morbidity and mortality rates [4].

Cessation of blood supply while excising the lungs from the donor for transplantation leads to ischemia and subsequent reperfusion during transplantation to the recipient. This causes a rapid host inflammatory response leading to injury and possible dysfunction of the transplanted organ [5]. Even though the reperfusion procedure induces additional damage to the donor lungs, this procedure is unavoidable, so as to prevent the lungs from facing irreversible ischemia after removal from the donor. Limiting or blocking oxygen supply followed by reperfusion leads to formation of reactive oxygen species (ROS) along with activation of innate immune responses and inflammatory influx [6]. The extent of damage to the transplanted lung due to ischemia reperfusion determines the survival rate and functionality of the lung in the recipient body.

Recent studies are now focusing on identifying methodologies to reduce or limit injury induced by ischemia and reperfusion. Studies devising therapeutic interventions that can alleviate tissue damage due to ischemia and reperfusion are underway [7–11]. This requires the understanding of molecular mechanisms and downstream signaling involved in the process of ischemia and reperfusion prior to injury. Recent studies have explored the possibilities of using miRNAs as a biomarker to predict or regulate injury induction in the context of ischemia reperfusion during lung transplantation [12, 13]. MicroRNAs include the group of small noncoding RNAs that regulate gene expressions by binding to the 3′untranslated region of target mRNAs thereby preventing their translation. miRNAs have gained huge biological and therapeutic importance due to their capacity to selectively and uniquely block specific mRNAs thereby controlling their expression pattern and function [14]. Microarrays from retinal, cerebral, and skeletal muscle IR in rodent models suggested differential expression of miRNAs such as mir-495, mir-207, mir-329, mir-152, mir-223, and mir-667 and their role in ischemia and reperfusion [15–17]. However, the role of miRNAs in lung ischemia reperfusion injury is not widely explored.

The current study is aimed at establishing a rat model of ischemic reperfusion in the lungs and evaluating the expression dynamics of miRNAs in rat lungs post ischemia reperfusion. This study identifies differentially expressing miRNAs through high-throughput analysis and contributes towards unveiling the complex molecular mechanism involved in ischemia reperfusion injury, especially in the lung model. This study also established an *in vitro* system of ischemia reperfusion by exposing type II alveolar epithelial cells to hypoxia and then replacing them to normal oxygenated conditions. We then validated one of the downregulated miRNAs, miR-18a-5p, in the *in vitro* model of IR. Through gene prediction and mutation analysis, we find and validate Galanin mRNA as a target for miR-18a-5p. Downregulation of miR-18a-5p upon IR leads to upregulation of Galanin, which then attenuates the deleterious effects of IR and leads to cell survival and proliferation. Perturbation of miR-18a-5p levels by mimics or inhibitor affects not only the ROS levels generated post-IR but also on the nuclear localization of transcription factor Nrf2, leading to transcriptional activation of cytoprotective genes. Thus, either the upregulation of Galanin or the downregulation of miR-18a-5p can alleviate the cellular injury caused by ischemia reperfusion and lead to cell survival and proliferation. Galanin, a neuropeptide, is known to play a protective role against insults by ischemia reperfusion in tissues such as the liver, brain, and heart, but its role in the lung has not been studied before [18–21]. The results from this study not only provide the miRNAs as possible biomarkers for early detection of IR-mediated lung injury but also provide a therapeutic intervention to prevent further cell death.

## 2. Materials and Methods

### 2.1. Animal Use and Ethical Approval

Sprague Dawley rats of clean grade were used for this work. Male rats were used which were 7-8 weeks old and weighed around 220-280 g at the time of the experiments. All the experiments were approved by the Animal Ethics Committee of the Chinese PLA General Hospital.

### 2.2. Surgery and Ischemia Reperfusion

Intraperitoneal injection of urethane was done for anesthesia, by tracheal intubation and connecting the animal to the ventilator (setting parameters were 70 times/min, tidal volume of 20 mL/kg, and respiration ratio of 1 : 1). Thoracotomy was performed on the fourth and fifth intercostal spaces of the left chest. The left hilum was peeled off and closed with a noninvasive blood vessel for 45 minutes. The blood flow was perfused, and the layers were sutured one after the other. The sham operation group only had the surgical access without clamping of the blood vessels.

### 2.3. Material Collections

Post-IR, the rats were euthanized, and lung tissues were dissected out. Part of the tissue was fixed by paraformaldehyde for sectioning for H&E staining, part of the tissue was frozen at −80°C for qPCR detection, and part was used for high-throughput sequencing of miRNA.

### 2.4. Hematoxylin and Eosin (H&E) Staining

#### 2.4.1. Tissue Embedding and Sectioning

Lung tissue was fixed at 4% paraformaldehyde for more than 24 hours. The tissue is taken out of the fixation solution, and a scalpel is used to smooth the tissue at the target site in the fume hood. The cut tissues are placed in the dehydration box.

#### 2.4.2. Dehydration

The dehydration box is placed into the hanging basket, and gradient alcohol is dewatered in order. The order was 75% alcohol for 4 h, 85% alcohol for 2 h, 90% alcohol for 2 h, 95% alcohol for 1 h, absolute ethanol I for 30 min, absolute ethanol II for 30 min, alcohol benzene for 5-10 min, xylene I for 5-10 min, xylene II for 5-10 min, wax I for 1 h, wax II for 1 h, and wax III for 1 h.

#### 2.4.3. Embedding

The soaked tissue is embedded using the embedding machine. Initially, the melted wax was placed in the embedding frame. Before the wax solidifies, the tissue from the dehydration box is removed and placed into the embedding frame according to the requirements of the embedding surface and the corresponding label is attached. The embedded tissue is then cooled in a refrigerator at −20°C. After the wax has solidified, the wax block is removed from the embedding box and trimmed.

#### 2.4.4. Slicing

The trimmed wax block is placed on a paraffin microtome and sliced to a thickness of 4 *μ*m. The sections were floated on 40°C warm water of the spreader to flatten the tissue. The tissue was picked up with a glass slide and placed in a 60°C oven to bake the slices.

#### 2.4.5. Staining

Paraffin sections of the lung tissue were dewaxed to water in the following order: xylene I for 20 min, xylene II for 20 min, absolute ethanol I for for10 min, anhydrous ethanol II for 10 min, 95% alcohol for 5 min, 90% alcohol for 5 min, 80% alcohol for 5 min, and 70% alcohol for 5 min and lastly in distilled water.

Sections were stained into Harris hematoxylin for 3-8 min, washed with tap water, 1% hydrochloric acid, and alcohol differentiated for several seconds, later rinsed with tap water, 0.6% ammonia water returned to blue, and rinsed with running water. Sections were stained in eosin solution for 1-3 min. The sections were then placed in order of 95% alcohol I for 5 min, 95% alcohol II for 5 min, absolute ethanol I for 5 min, anhydrous ethanol II for 5 min, xylene I for 5 min, and xylene II for 5 min for dehydration, and as the section became transparent, they are taken out from xylene. The sections are dried and sealed with neutral gum.

### 2.5. Cell Lines

Rat type II alveolar epithelial cells were used for the *in vitro* model IR. After plating the cells, 70% confluency was reached after 24 hours. The cells were hypoxic treated for 3 hours and then reoxygenated for 24 h. Another set of cells was left without any treatment. After 24 hours, the supernatant was discarded, washed with PBS, and proceeded with RNA extraction.

### 2.6. RNA Extraction

RNA extraction for all the samples was carried out using TRIZOL and as per the mentioned protocol.

### 2.7. miRNA Sequencing and Analysis

#### 2.7.1. Library Construction

After the RNA samples are qualified for library preparation, the small RNA Sample Prep Kit was used to construct the library. The special structure of the 3′and 5′ends of the small RNA (the 5′end has a complete phosphate group and the 3′end has a hydroxyl group) was used. Total RNA was used, and adapters are directly added to the ends of small RNA, and then, cDNA is synthesized by reverse transcription. After the PCR amplification, the target DNA fragments are separated by PAGE gel electrophoresis, and the cDNA library is obtained by cutting the gel (Supplementary Figure 1A).

#### 2.7.2. Library Inspection

After the library construction is completed, Qubit 2.0 is used for preliminary quantification, the library was diluted to 1 ng/*μ*L, and then, the Agilent 2100 was used to detect the insert size of the library. After the insert size is as expected, the qPCR method was used to determine the effective concentration of the library.

#### 2.7.3. HiSeq Sequencing

After the library is qualified, different libraries are pooled according to the requirements of effective concentration and target offline data volume, and then, HiSeq/MiSeq sequencing is performed.

### 2.8. Bioinformatics Analysis

Given that the used species is a rodent and has a reference genome, an animal small RNA analysis method is used as described in Supplementary Figure 1B.

### 2.9. First-Strand cDNA Synthesis

MMLV RT kit (ELK Biotechnology EQ002) was used for first-strand cDNA synthesis. The manufacturer's protocol was followed.

### 2.10. Real-Time PCR

Real-time PCR was performed on a StepOne™ Real-Time PCR instrument from Life Technologies. Each sample was made in 3 replicates using the EnTurbo™ SYBR Green PCR SuperMix Kit (ELK Biotechnology, EQ001).

Data analysis was done by the *ΔΔ*CT method.

(1) $$\begin{array}{c} A=\text{CT }\left(\text{target gene},\text{experimental sample}\right)-\text{CT }\left(\text{internal standard gene},\text{experimental sample}\right),\\ B=\text{CT }\left(\text{target gene},\text{control sample}\right)-\text{CT }\left(\text{internal standard gene},\text{control sample}\right),\\ K=A-B,\\ \text{Expression multiple}=2^{-K}. \end{array}$$

### 2.11. Prediction of rno-miR-18a-5p and Gal Interaction

The sequence of rno-miR-18a-5p is as follows: UAAGGUGCAUCUAGUGCAGAUAG.

The sequence of Gal (ENSRNOT00000020425.5) 3′UTR is GACCACACCCACTGTGCACCTGTGTCCTCTGCTATAATTTAAAGTCATTCTAGGCTAAAAAGAATCTTCCGCCAACTCCTCAAGCCAACACTTTGTTCTCTGCTTTGATGCTGAGTTATTACAATTAAGATGTTTTGATTGGAGTAATTATATTGTGTGACATAATAAAAACTAGCAAGTAACTGGACTGTTTGGTTCTTCTATGCTGCGTCTATCACTGCCACCTCCTGTGTAGTTTATTGTATTTTGTGTGTGTGTGTGTGTGTGTGTGTGTGTGTGTGTGTGTGTGCACATGCATGTGTGCACCTTGTGTACTGAACTC.

Predicting the combination of miR-18a-5p and 3′UTR of Gal mRNA, the prediction results are shown in Supplementary Figure 2.

### 2.12. Dual Luciferase Assay

The predicted wild and mutated sequence of about 200 bp upstream and downstream of the interaction site with rno-miR-18a-5p (only mutation predicted interaction site) of the Gal 3′UTR was synthesized and was inserted into the luciferase reporter gene vector pmirGLO. HEK293T cells were transfected with the miR-18a-5p mimics or control along with the luciferase constructs using the transfection reagent Lipofectamine 2000 (Invitrogen). 48 hours after transfection, cells were lysed and chemiluminescence readings were taken with the Dual Luciferase Kit (Beyotime) and the instrument. Renilla luciferase was used as an internal reference, and the RLU value measured by firefly luciferase is divided by the RLU value measured by Renilla luciferase.

### 2.13. Primers

Primers used for quantitative analysis of miRNAs were synthesized by Wuhan GeneCreate Biological Engineering Co., Ltd. Primer sequences are shown in Table 1.

### 2.14. ELISA to Detect Serum Inflammatory Factors

The kits shown in Table 2 were utilized for detection of proinflammatory molecules from rat blood serum. Standards were derived for each molecule and the line equations were used to deduce the concentrations in each sample, according to the kit protocol.

### 2.15. Cell Survival Assay

AT2 cells were treated with hypoxia (3 h) and reoxygenation for periods of 0, 24, 48, and 72 h along with miR-18a-5p mimics or inhibitor and respective controls. CCK8 solution was then added to each well and incubated for 1-4 hours. The absorbance was taken at 450 nm in a microplate reader.

### 2.16. Apoptosis Assay

AT2 cells were treated with hypoxia (3 h) and reoxygenation for 24 h along with miR-18a-5p mimics or inhibitor and respective controls. For the apoptosis assay, cells at a concentration of 1 × 10^6^/mL were taken and centrifuged at 1000 rpm for 5 min. After two PBS washes, the cells were suspended in 100 *μ*L of binding buffer, Annexin V-FITC, and incubated for 15 min. The cells are transferred to a flow detection tube, and 400 *μ*L of PBS with 1 *μ*L PI is added to each sample and incubated for 2 min. The cells were then passed through the Attune NxT sonic focusing flow cytometer. Data was then analyzed through the flow cytometric analysis software.

### 2.17. Flow Cytometer Detection of ROS

AT2 cells were treated with hypoxia (3 h) and reoxygenation for 24 h along with miR-18a-5p mimics or inhibitor and respective controls. DCFH-DA probe was diluted with serum-free medium to a final concentration of 10 *μ*mol/L. The cell culture medium is removed and 1 mL of diluted DCFH-DA to each six-well plate and incubated at 37°C for 20 min. After washes, the fluorescence was detected using flow cytometry. Data was then analyzed through the flow cytometric analysis software.

### 2.18. Confocal Imaging

For calcium imaging, Fluo-4 AM (Biyuntian, S1060) mother liquor was diluted with PBS to a working solution of 0.5-5 *μ*M. AT2 cells treated with hypoxia/reoxygenation with miR-18a-5p mimics or inhibitor, and respective controls were washed with PBS and Fluo-4 AM was added to cover the cells. The solution was incubated with cells for 10-60 min at 20-37°C. After washing with PBS, the cells were further incubated for 20-30 min to ensure that Fluo-4 AM is completely converted into Fluo-4 in the cells. The fluorescence was then detected with a laser confocal microscope (PerkinElmer & Olympus, UltraVIEW VoX & IX81).

For Nrf2 immunostaining, cells were fixed in 4% paraformaldehyde for 10-15 min and then washed with PBS (3x). After permeabilization with 0.2% Triton X-100 for 10 minutes, the cells were blocked with 10% goat serum for 30 min at 37°C. Primary antibody (Nrf2, Abcam) was made in 5% goat serum and incubated with the fixed cells overnight. Post primary antibody incubation, 3x PBS washes were given and the cells were incubated with secondary antibody (prepared in 5% goat serum) at 37°C for 1 h. The cells were then incubated with nuclear stain DAPI and sealed on glass slides. The fluorescence was then detected with a laser confocal microscope (PerkinElmer & Olympus, UltraVIEW VoX & IX81).

### 2.19. Immunoblotting

AT2 cells were treated with hypoxia (3 h) and reoxygenation for 24 h along with miR-18a-5p mimics or inhibitor and respective controls. Cells were then lysed, and protein estimation was done using the BCA kit. Appropriate amount of total protein was taken for immunoblotting. Lysates were electrophoresed on 12% resolving gel for 45 min and transferred on to a PVDF membrane at 1.5 mA/cm^2^ gel volume for 1.5 h. The immunoblots were probed for respective primary and secondary antibodies and developed using the ECL luminescent solution in a dark room.

### 2.20. Statistical Analysis

The data were analyzed using Microsoft Excel 2007 software (https://www.microsoft.com; Microsoft Corporation, Redmond, WA, USA). All data are presented as the mean ± standard deviation. For comparisons between two groups, statistically significant differences between means were identified by paired Student's *t*-test. For multiple comparisons, the significance was determined by simple one-way analysis of variance followed by Tukey's post hoc test. *P* < 0.05 was considered to indicate a statistically significant difference.

## 3. Results

### 3.1. Establishing Ischemia Reperfusion Model

Male Sprague Dawley rats aged 7 to 8 weeks were used for establishing ischemia reperfusion model by hilar occlusion of the lung. Model development and validation of injury were then demonstrated using ELISA and histology.

### 3.2. ELISA and Histology

**Figure 1:**
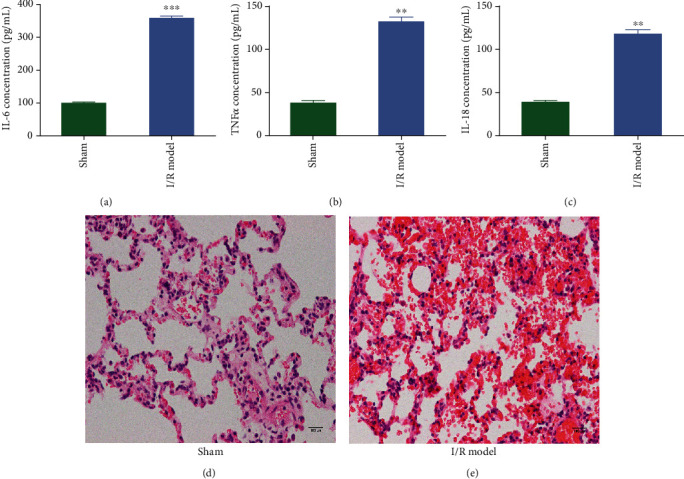
Proinflammatory response and injury in rat model of IR. Quantitative analysis of proinflammatory molecules (a) interleukin-6 (IL-6), (b) tumor necrosis factor-alpha (TNF-*α*), and (c) interleukin-18 (IL-18) from sham-operated (sham) or ischemia reperfusion (I/R model) rat serum using ELISA. Values were quantified based on a standard obtained with known concentrations of each molecule (*n* = 3, ±SEM, ^∗^*P* < 0.05). (d) Representative histological section of hematoxylin and eosin- (H&E-) stained lung tissue from sham-operated (sham) rat models. (e) Representative histological section of hematoxylin and eosin- (H&E-) stained lung tissue from ischemia reperfusion (I/R model) treated rat models.

Enzyme-linked serum immunosorbent assay (ELISA) was performed to determine the variation in the levels of serum inflammatory factors in IR injury rats and sham models. Compared with the sham operation group, the levels of interleukin-6 (IL-6), interleukin-18 (IL-18), and tumor necrosis factor-*α* (TNF-*α*) in the serum of the I/R model group were significantly higher (Figure 1). This increase of serum inflammatory factors in blood after ischemia reperfusion proves that the rat ischemia reperfusion model was successfully established. IL-6 level in rat serum after IR induction was 355.9 ± 14.1 pg/mL compared to 97.2 ± 5.7 pg/mL in sham models (Figure 1(a)). Similarly, IL-18 level in rat serum was 116.3 ± 9.77 in IR models as compared to 38.4 ± 3.61 in sham models (Figure 1(c)). TNF-*α* levels in IR models were 131.9 ± 9.8 against 3.42 ± 5.6 in sham models (Figure 1(b)). This hike in the inflammatory cascade molecules after IR injury induction confirms successful model development [22, 23]. The gross observation of the lungs of the sham-operated group appeared normal while in the IR group the lung volume was slightly increased compared to sham models. The H&E-stained lung tissue of the sham group appeared normal with no swelling and minimal inflammatory cell infiltration. The alveolar cavity and wall were smooth and normal with no thickening (Figure 1(d)), whereas the H&E-stained lung tissue sections of the ischemia reperfusion group appeared dark red with distorted alveolar geometry (Figure 1(e)). Histology staining displayed huge infiltration of inflammatory cells with vascular congestion and pulmonary edema in the IR group. The alveolar septa appeared to be thicker than normal. These observations can be considered as a hallmark for IR-induced injury [24].

### 3.3. High-Throughput Analysis of miRNAs in a Rat Model of Lung Ischemia Reperfusion

**Figure 2:**
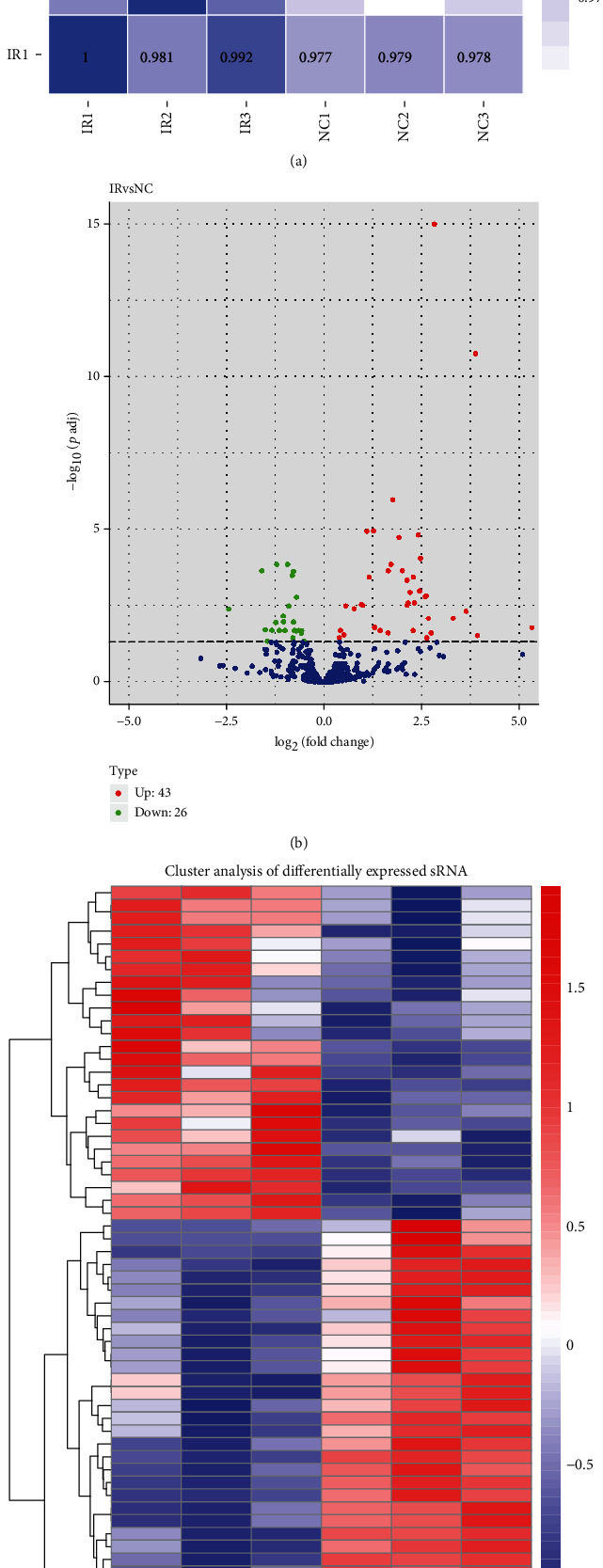
High-throughput analysis of differentially expressed miRNA in NC vs. IR rat models. (a) Pearson's correlation analysis between all six set of samples (NC 1-3, IR 1-3) used for high-throughput sequencing of miRNAs. (b) Volcano plot showing the distribution of miRNAs that are significantly upregulated (red) and downregulated (green) or showed no change (blue) on ischemia reperfusion (IR) as compared to sham-operated (NC) rat models. (c) Hierarchical clustering of miRNAs vs. samples where rows represent the clustering of miRNAs and columns represent the clustering of samples. As the miRNA abundance ratio changes from small to large, the heat map color shows a corresponding red-white-blue change.

After confirming IR injury through histology in rat lungs, the miRNA signature in these IR-induced rat lung tissues was studied in comparison to that in sham models. Total RNA was extracted from the frozen lung tissue, and a miRNA library was built from good quality of RNA. The insert size of the library was confirmed by Agilent 2100, and it was quantified using Qubit 2.0. The library for each sample was then subjected to HiSeq sequencing. To check for the reproducibility of the replicates and relation between the two groups, Pearson's correlation analysis was done on all six samples (Figure 2(a)). The biological replicates showed high correlation with each other (IR1 vs. IR2 vs. IR3) but lower correlation among the two groups (IR1 vs. NC1). This showed that the replicates were reproducible and also that the IR treatment caused a change in the expression profile of miRNAs between the groups, reflected by the lower correlation between the groups (IR vs. NC). Differential expression analysis of the miRNAs revealed 69 significantly altered miRNAs (*P* value < 0.05) in the IR model vs. the sham model (Supplementary Table S1). The volcano plot clearly shows the distribution of these differentially expressed miRNAs; 43 different miRNAs are upregulated labelled in red and 26 miRNAs are downregulated labelled in green (Figure 2(b)). These differentially expressed miRNAs also cluster together as shown by the hierarchical cluster analysis (Figure 2(c)); those that are downregulated are more closely related and are similar to each other as compared to those that are upregulated. This global analysis of miRNAs on lung IR injury shows that there is a miRNA-mediated response which plays an important role in IR and the downstream pathophysiology. There are several miRNAs already reported to be involved in ischemia reperfusion [13, 17, 25–32] in various tissue types. But since we are establishing a new rat model for lung ischemia reperfusion, we decided to take an unbiased, global approach to look at the miRNA signature on ischemia reperfusion. Towards this regard, we decided to validate the top three candidates in each category (upregulated and downregulated) (Table 3) of the high-throughput analysis by quantitative qPCR in the same rat lung IR tissue vs. sham model.

### 3.4. Validation of Differentially Expressed miRNAs by qPCR

#### 3.4.1. *In Vivo* System of Ischemia Reperfusion Injury

**Figure 3:**
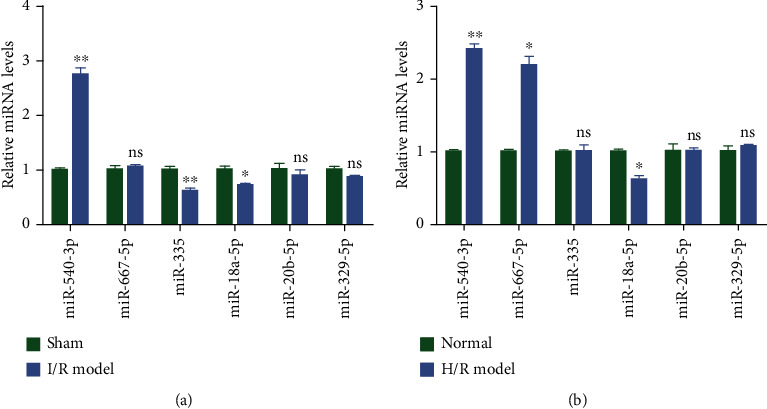
Validation of differentially expressed miRNAs by qPCR in rat model and *in vitro* of IR. (a) Quantitative validation of top three upregulated and three downregulated miRNAs by qPCR from rat lung tissue with ischemia reperfusion (I/R model) or sham operation (sham) for rno-miR-540-3p, rno-miR-329-5p, rno-miR-667-5p, rno-miR-335, rno-miR-18a-5p, and rno-miR-20b-5p. (b) Quantitative validation of top three upregulated and three downregulated miRNAs by qPCR from AT2 cells with hypoxia treatment and reoxygenation (H/R model) or no treatment (normal) for rno-miR-540-3p, rno-miR-329-5p, rno-miR-667-5p, rno-miR-335, rno-miR-18a-5p, and rno-miR-20b-5p. U6 snRNA was used as an internal control. All values are normalized to those obtained from NC or WT samples (*n* = 3, unpaired Student's *t*-test, ±SEM, ^∗^*P* < 0.05).

The top three genes that were significantly upregulated in IR models (miR-667-5p, miR-329-5p, and miR-540-3p) and the top three genes that were significantly downregulated in IR injury models (miR-335, miR-18a-5p, and miR-20b-5p) obtained through the high-throughput sequencing experiment were then validated using qPCR using specific primers (Figure 3 and Table 1). Out of the three genes identified as upregulated in the high-throughput sequencing experiment, rno-miR-540-3p displayed significant upregulation in the IR model (I/R model) when validated using qPCR as observed in the high-throughput sequencing study while miR-329-5p and miR-667-5p of IR models did not show significant changes in their expression pattern when compared to sham models (sham) (Figure 3(a)), whereas for the genes identified to be downregulated in the high-throughput sequencing experiment, rno-miR-335 and rno-miR-18a-5p were significantly downregulated in IR models upon validation with qPCR while miR-20b-5p did not show any significant change in expression when compared to the sham model (Figure 3(a)). rno-miR-540-3p displayed almost a twofold upregulation while rno-miR-335 and rno-miR-18a-5p were downregulated by approximately half a fold.

#### 3.4.2. *In Vitro* System to Demonstrate Ischemia Reperfusion Injury

An *in vitro* cell-based culture system using rat type II alveolar epithelial (AT2) cells with hypoxia treatment followed by reoxygenation was evaluated for its functional efficiency as ischemic reperfusion model. The *in vitro* alveolar cells treated for simulating IR model were compared with normal alveolar cells and evaluated for the expression pattern of those miRNAs identified using the high-throughput sequencing studies by using qPCR (Figure 3).

Out of the three miRNAs identified as upregulated in the high-throughput sequencing experiment, rno-miR-540-3p and rno-miR-667-5p showed more than 1.5-fold upregulation in hypoxia/reoxygenation (H/R model)-treated alveolar cells when compared to alveolar cells cultured in normal conditions (Figure 3(b)), whereas rno-miR-329-5p of H/R-treated cells did not show significant changes in the expression pattern when compared to normal cells (normal) (Figure 3(b)). In the case of miRNAs identified to be downregulated in high-throughput sequencing experiment, rno-miR-18a-5p was significantly downregulated in the H/R *in vitro* model (Figure 3(b)), while rno-miR-335 and rno-miR-20b-5p did not show any significant change in expression when compared to normal cells (Figure 3(b)).

### 3.5. miR-18a-5p Target Gene Prediction and Gal mRNA Analysis

**Figure 4:**
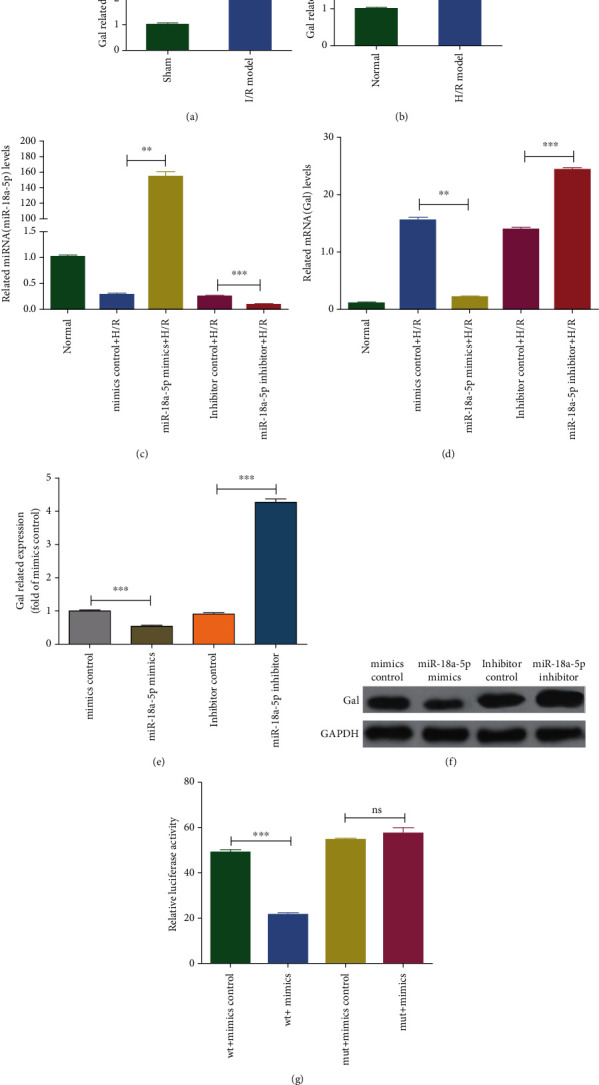
Evaluation of GAL mRNA levels on IR and its association with miR-18a-5p. (a) Quantitative validation of GAL mRNA by quantitative PCR from rat lung tissue with ischemia reperfusion (I/R model) or sham operation (sham). (b) Quantitative validation of GAL mRNA by qPCR from AT2 cells with hypoxia treatment and reoxygenation (H/R model) or no treatment (normal). GAPDH mRNA was used as an internal control. All values are normalized to those obtained from sham or normal samples (*n* = 3, unpaired Student's *t*-test, ±SEM, ^∗^*P* < 0.05). (c) Quantitative analysis of miR-18a-5p levels on treatment of AT2 with hypoxia and reoxygenation (H/R) transfected with miR-18a-5p mimics or inhibitor or no treatment (normal). U6 snRNA was used as an internal control. All values are normalized to those obtained from normal samples. (d) Quantitative analysis of GAL mRNA levels on treatment of AT2 cells with hypoxia and reoxygenation (H/R) in the presence of miR-18a-5p mimics or inhibitor or no treatment (WT). GAPDH mRNA was used as an internal control. All values are normalized to those obtained from normal samples (*n* = 3, unpaired Student's *t*-test, ±SEM, ^∗^*P* < 0.05). (e) Quantitative analysis of GAL mRNA levels on treatment of AT2 cells transfected with miR-18a-5p mimics or inhibitor. GAPDH mRNA was used as an internal control. All values are normalized to those obtained from transfected with mimic control samples (*n* = 3, unpaired Student's *t*-test, ±SEM, ^∗^*P* < 0.05). (f) Representative western blotting images for Gal transfected with miR-18a-5p mimics or inhibitor or respective controls. (g) Quantitative analysis of relative luciferase activity of pmirGLO-GAL-wt or pmirGLO-GAL-mut transfected in HEK293T cells in the presence of miR-18a-5p mimics (*n* = 3, unpaired Student's *t*-test, ±SEM, ^∗^*P* < 0.05).

Of the three downregulated miRNAs that were validated by using both the *in vivo* and *in vitro* model of ischemic reperfusion injury, rno-miR-18a-5p showed a consistent reduction on IR as compared to sham model. So far, there are no reports on the role of rno-miR-18a-5p in ischemic reperfusion especially in the lung. Thus, the novel role of this miRNA was followed in this study. As miRNAs are known to target mRNAs leading to their translation repression, the next aim was to look at the mRNAs that are targeted by this miRNA. From the miRNA sequencing results and target gene analysis (Supplementary Table S2), it is known that rno-miR-18a-5p targeted 13 genes, of which 8 are upregulated and Galanin (Gal) was found to be significantly upregulated. Gal is a known neuropeptide found in most parts of the brain and in the peripheral nervous system [33]. It is known to play a role in secretion of hypothalamic-pituitary hormones and nociception and controls the sleep cycle [21]. Gal expression has shown to undergo ischemia-related increase in the hippocampal neurons and protects these neurons from damage by opening up potassium channels [33]. Similarly, treatment with Gal fragments increased the cell viability and inhibited cell apoptosis and excessive mitochondrial ROS in cardiomyoblasts exposed to hypoxic stress [20, 34]. Hence, Gal was a valid candidate for our study of lung ischemic reperfusion. Additionally, there are no known reports of miRNA-mediated Gal mRNA translation regulation. Thus, the regulation of Gal expression via rno-miR-18a-5p under lung ischemic reperfusion was assessed. For the same, the expression of Gal mRNA on ischemic reperfusion in the rat lung tissue and from the H/R model of AT2 cells was evaluated (Figure 4). A statistically significant increase in the levels of Gal mRNA in the IR lung tissue as compared to the sham model when normalized against the housekeeping GAPDH mRNA was observed (Figure 4(a)). Similarly, an increase in the expression of Gal mRNA in the H/R-treated AT2 cells as compared to the WT cells was observed (Figure 4(b)). To assert the role of rno-miR-18a-5p in regulating the levels of Gal mRNA, the levels of rno-miR-18a-5p were perturbed by either overexpressing the mimics or inhibitor of rno-miR-18a-5p during H/R *in vitro* model of AT2 cells. The levels of rno-miR-18a-5p were evaluated by qPCR in all the conditions, and the levels were significantly higher in the presence of the mimics and significantly lower in the presence of the inhibitor (Figure 4(c)). The levels of Gal mRNA were then assessed in the presence of the rno-miR-18a-5p mimics or inhibitor. The levels of Gal mRNA decreased in the presence of mimics even on H/R conditions. Interestingly, the levels of Gal mRNA increased in the presence of inhibitor and H/R conditions as compared to control conditions (Figure 4(d)) thus implying a role of rno-miR-18a-5p in H/R-mediated increase in Gal mRNA expression. Furthermore, miRNA inhibitor and mimics were transfected into AT2 cells in normoxic environment, and the expression of Gal mRNA and protein in the rno-miR-18a-5p mimic group decreased, while the result in the inhibitor group was opposite to that in the mimic group (Figures 4(e) and 4(f)). The association Gal mRNA and rno-miR-18a-5p was studied by analyzing the 3′UTR of the Gal mRNA for miRNA binding sites (described in Materials and Methods). A perfect complementarity was observed in the seed region of the miRNA (2-7 nt) and the 3′UTR region of Gal mRNA. Luciferase constructs containing the 3′UTR with or without miRNA binding site mutation were transfected in the HEK293T cells along with rno-miR-18a-5p mimics. A reduced luciferase activity was observed in the presence of the miR-18a-5p mimics as compared to control mimics. Corroborating this result, no change in the luciferase activity was observed when the mutant form of 3′UTR was used as compared to the mutant mimic control (Figure 4(g)).

### 3.6. Role of Gal in Ischemia Reperfusion-Mediated ROS Generation and Apoptosis

**Figure 5:**
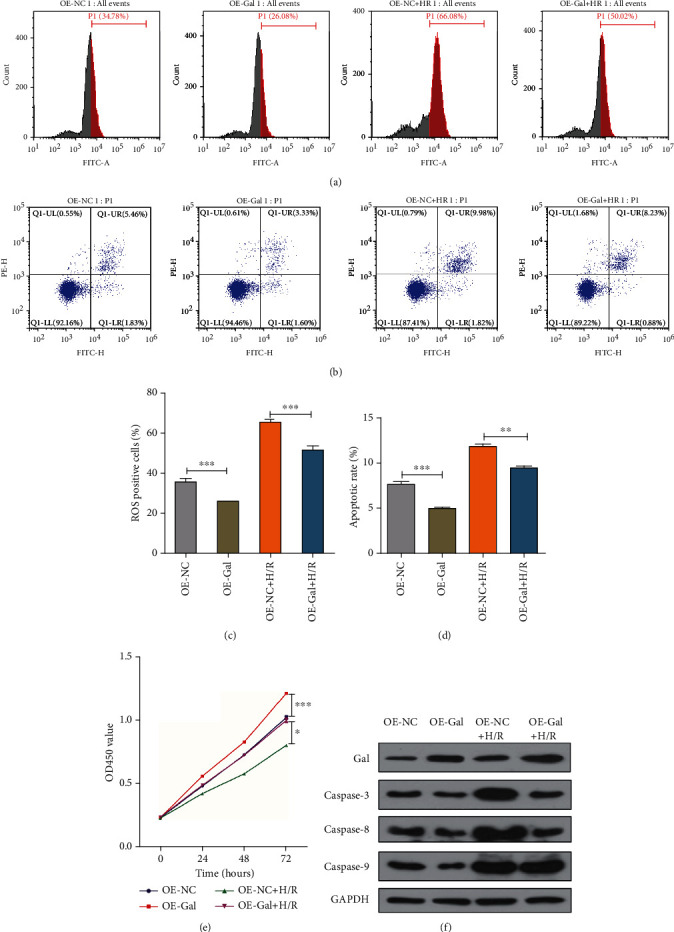
The role of Gal in lung injury induced by hypoxia/reoxygenation. (a, c) Flow cytometry of rat AT2 surface probed with dye DCFH-DA for detection of ROS. (b, d) Flow cytometry of rat AT2 cells with Annexin V-FITC-PI for detection of apoptosis (*n* = 3, unpaired Student's *t*-test, ±SEM). (e) Cell proliferation analysis from 0 h up to 72 h for rat AT2 cells treated with hypoxia/reoxygenation and with vector for overexpression of Gal. (f) Detection of Gal, caspase-3, caspase-8, and caspase-9, the key molecules of Gal-related apoptosis signaling pathway by western blot in rat AT2 cells transfected with miR-18a-5p mimics or inhibitor or respective controls.

Pulmonary ischemia reperfusion often leads to the increase of ROS in lung tissue, which can lead to apoptosis. We observed that overexpression of Gal inhibited the level of ROS and alleviated the increase of ROS induced by hypoxia reoxygenation (Figures 5(a)). Apoptosis was detected by Annexin V-FITC-PI staining through flow cytometry assay. The results showed that the level of apoptosis was consistent with the increase of ROS; overexpression of Gal inhibited the apoptosis induced by H/R (Figures 5(b) and 5(d)). By detection of caspase-3, caspase-8, and caspase-9, the key molecules of Gal-related apoptosis signaling pathway by western blot, we found that the expression of caspase-3, caspase-8, and caspase-9 was decreased after Gal overexpression (Figure 5(f)). Moreover, we detected the proliferation of AT2 cells at 0, 24, 48, and 72 hours by CCK8 assay. The results showed that overexpression of gal promoted the growth of AT2 cells and alleviated the inhibition of H/R on cell viability (Figure 5(e)).

### 3.7. Role of miR-18a-5p in IR-Mediated Apoptosis

**Figure 6:**
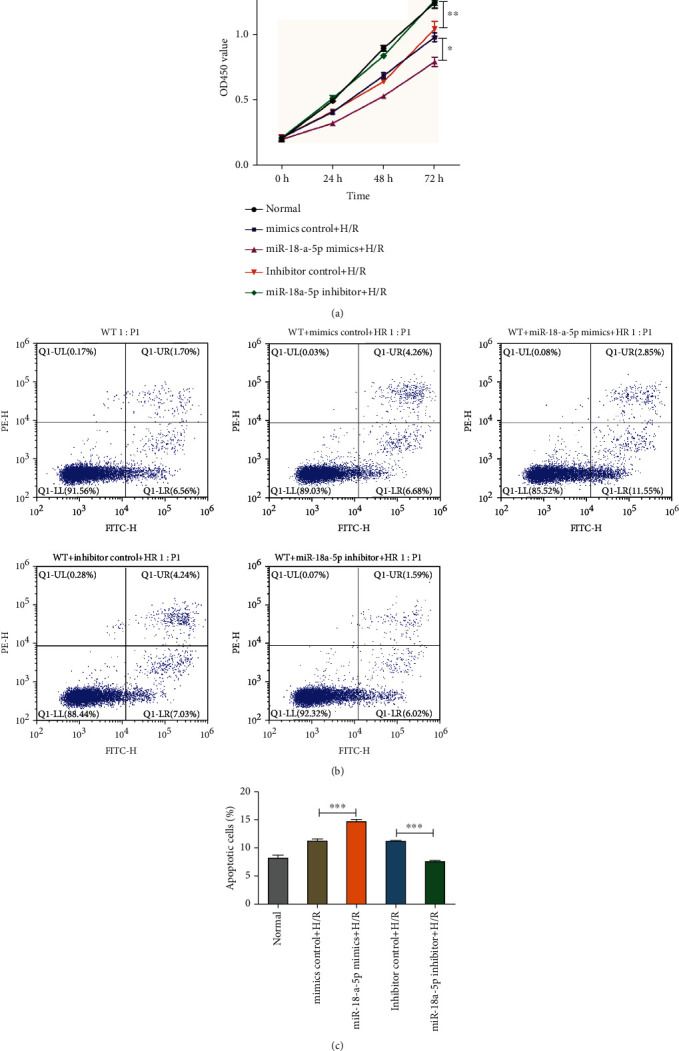
Effect of hypoxia/reoxygenation and rno-miR-18a-5p on cell proliferation and apoptosis. (a) Cell proliferation analysis from 0 h up to 72 h for AT2 cells treated with hypoxia/reoxygenation and with miR-18a-5p mimics and inhibitor. (b) Panels 2 and 3 show the profiles of AT2 cells costained with FITC-Annexin V and propidium iodide for no treatment (WT), hypoxia/reoxygenation (H/R) transfected with mimics control or with miR-18a-5p mimics. Panels 4 and 5 show the profile for cells costained with FTCI-Annexin V and propidium iodide hypoxia/reoxygenation (H/R) transfected with inhibitor control or with miR-18a-5p inhibitor. (c) Quantitative analysis of the percent apoptotic cells (early+late) from the FITC-PI profiles after treatment with hypoxia/reoxygenation, in the presence of miR18a-5p mimics and inhibitor (*n* = 3, unpaired Student's *t*-test, ±SEM).

Ischemia reperfusion injury is known to cause inflammation and generation of ROS leading to cell death [35–37]. The cell proliferation response to H/R was studied in the AT2 in the presence of rno-miR-18a-5p mimics or inhibitor (Figure 6(a)). Using the CCK8 cell proliferation kit, the absorbance gradually reduced when AT2 cells were treated with H/R as compared to no treatment (normal). This reduction was accentuated in the presence of rno-miR-18a-5p mimics but abrogated and recovered to the normal levels when rno-miR-18a-5p inhibitor was used. To ascertain the cause of reduced cell proliferation, cells treated with H/R and rno-miR-18a-5p mimics or inhibitor were stained with Annexin V and PI. While H/R treatment leads to the increase in the percent of early and late apoptotic cells as compared to no treatment (normal), the overexpression of rno-miR-18a-5p mimics further increased this percentage of apoptotic cells (Figures 6(b) and 6(c)). Interestingly, the overexpression of rno-miR-18a-5p inhibitor reduced the percentage of apoptotic cells even after treatment with H/R (Figures 6(b) and 6(c)), implying a causative role of rno-miR-18a-5p in mediating apoptosis and cell death due to H/R.

### 3.8. Role of miR-18a-5p in Ischemia Reperfusion-Mediated ROS Generation

**Figure 7:**
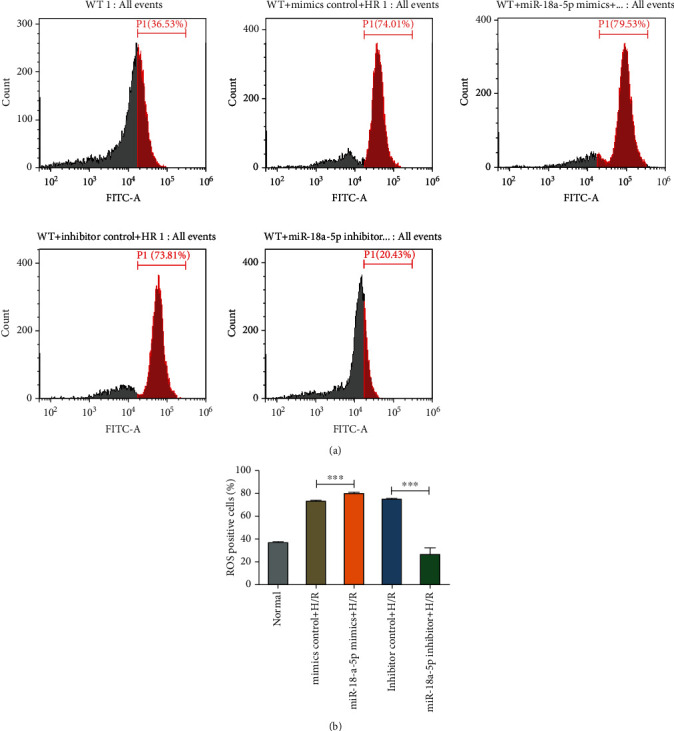
Effect of hypoxia/reoxygenation and miR-18a-5p on ROS generation. (a) Flow cytometry of rat AT2 surface probed with dye DCFH-DA for detection of ROS. Middle panels show the ROS-positive cells (red histogram) treated with hypoxia/reoxygenation (WT+H/R) and transfected with miR-18a-5p mimics. Lower panels show the ROS-positive cells and treated with hypoxia/reoxygenation (WT+H/R) and transfected with miR-18a-5p inhibitor. (b) Quantitative analysis of ROS-positive cells obtained from the flow cytometric assay for AT2 cells treated with hypoxia/reoxygenation (H/R) and transfected with miR-18a-5p mimics or inhibitor (*n* = 3, unpaired Student's *t*-test, ±SEM).

A decrease in the rate of cell proliferation and an increase in the apoptotic cell number on H/R were observed (Figure 6) which would be due to the ROS generation in the H/R-treated cells. To check the same, a fluorogenic dye DCFH-DA was used to probe ROS molecules in AT2 cells treated with H/R and in the presence of rno-miR-18a-5p mimics or inhibitor (Figure 7). Flow cytometric analysis showed that H/R treatment increased the ROS-positive cells as compared to no treatment, whereas overexpression of rno-miR-18a-5p mimics further increased this number. Intriguingly, rno-miR-18a-5p inhibitor led to reduction in the ROS-positive cells even after treatment of H/R, implying a role of rno-miR-18-5p in ROS generation (Figures 7(a) and 7(b)).

### 3.9. Role of miR-18a-5p in Calcium Overload and Nrf2 Localization

**Figure 8:**
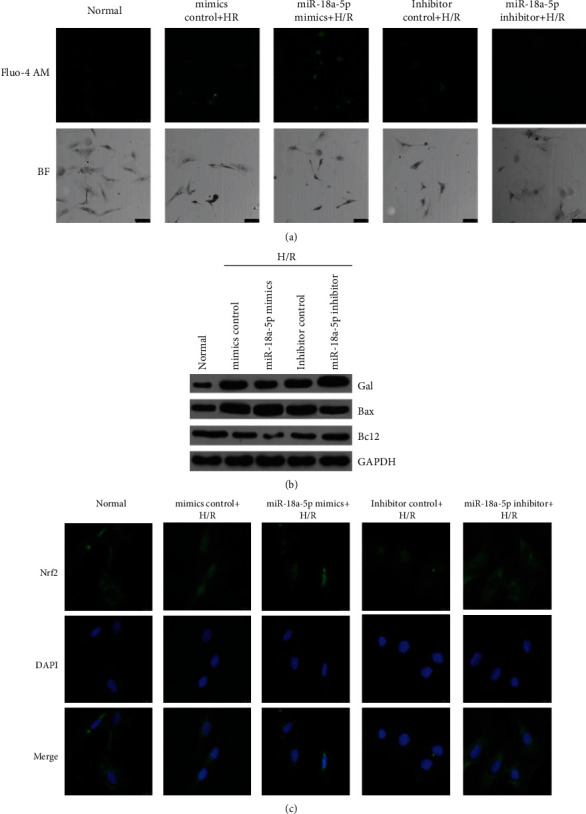
Effect of hypoxia/reoxygenation and rno-miR-18a-5p on intracellular calcium, apoptotic proteins, and localization of Nrf2. (a) Representative confocal images of intracellular calcium probed by Fluo-4 AM dye for AT2 cells treated with hypoxia/reoxygenation and transfected with miR-18a-5p mimics or inhibitor or respective controls. The lower panels are brightfield images of the same field as of the confocal images. (b) Representative western blotting images for Gal, Bax, Bcl2, and GAPDH for AT2 cells treated with hypoxia/reoxygenation and transfected with miR-18a-5p mimics or inhibitor or respective controls. (c) Representative confocal images of AT2 cells for transcription factor Nrf2 and nuclear marker DAPI, after treatment with H/R and transfected with miR-18a-5p mimics or inhibitor or respective controls.

Ischemic reperfusion is known to lead to intracellular calcium accumulation and ROS generation leading to activation of the proapoptotic pathways [36–39]. Through flow cytometric analysis, it was shown that IR leads to ROS generation and increase in apoptosis (Figures 5–7). Next, the levels of calcium and apoptotic proteins after H/R treatment in AT2 in the presence of rno-miR-18a-5p mimics or inhibitor were assessed (Figure 8). Fluo-4 AM dye was used to probe for intracellular calcium in AT2 cells after H/R treatment with rno-miR18a-5p mimics or inhibitor. The cells showed increased fluorescence on H/R treatment which further increased on overexpression of rno-miR-18a-5p mimics but decreased on overexpression on rno-miR-18a-5p inhibitor (Figure 8(a)) clearly suggesting a role of rno-miR-18a-5p in calcium overload in the cells post-H/R treatment.

To study the effect on the apoptotic proteins, Bcl-2 and BAX, immunoblotting analysis was done for these proteins after treatment of AT2 cells with H/R and in the presence of rno-miR-18a-5p mimics or inhibitor. H/R treatment leads to increase in the protein levels of GAL (Figure 8(b)) which corroborates with the increase in Gal mRNA levels (Figure 8(b)), but also show an increase in the proapoptotic protein BAX (Figure 8(b)). While the levels of GAL protein are reduced in the presence of miR-18a-5p mimics, the levels of BAX stay high as compared to control cells (WT). Interestingly, the levels of Bcl-2, antiapoptotic protein, are reduced on H/R treatment and go further lower on overexpression of rno-miR-18a-5p mimics. The presence of miR-18a-5p inhibitor during the H/R treatment increases the level of GAL more than that of just the H/R treatment, whereas the levels of both BAX and Bcl-2 are brought back to the WT levels when miR-18a-5p inhibitor is used during the H/R treatment. Thus, miR-18a-5p clearly plays a role in mediating an apoptotic response to ischemia reperfusion.

To elucidate the transcriptional role in the anti-inflammatory response and the change in miRNA expression, we decided to look at the Nrf2/ARE (antioxidant responsive element) pathway, which is known to be activated during oxidative stress [40, 41]. Nrf2 is a cytoplasmic protein, but upon increase in cellular ROS, it is known to be localized to the nucleus and activates the transcription of cytoprotective genes [42–44]. Thus, the localization of Nrf2 was observed on H/R treatment in the AT2 cells by confocal microscopy (Figure 8(c)). The localization of Nrf2 to the nucleus increased on H/R treatment, but it increased further in the presence of miR-18a-5p mimics. The increase of Nrf2 in the nucleus of the AT2 cells was blocked when the miR-18a-5p inhibitor was used during the H/R treatment. This thus indicates the role of miR-18a-5p in Nrf2-mediated transcription activation in response to ischemia reperfusion.

## 4. Discussion

Ischemia reperfusion injury (IRI) is commonly occurring pathological condition post organ transplantation. Lung IRI is known to occur after lung transplantation or cardiac bypass surgery, because both the surgeries require blocking of blood supply for up to 60 minutes and then reintroduction of the blood supply. This lack of blood and then reperfusion cause the tissue to experience dearth of oxygen leading to downstream signaling activating a severe inflammatory response. This inflammatory response leads to injury to the tissues causing cell death and organ failure [4, 45]. Yet, the pathogenesis and mechanistic insights of lung ischemia reperfusion injury are far less known. Thus, it becomes important to understand the molecular mechanism and cellular signaling involved in the process of lung ischemia reperfusion injury.

To study lung IRI, we established a rat model system. As reported, lung IRI leads to downstream activation of several kinases such as MAPK, c-Jun terminal kinases, and p38, thus promoting the secretion of several proinflammatory cytokines such as tumor necrosis factor-*α* [22, 46]. TNF-*α* is known to be the earliest inflammatory mediator that regulates the expression of various inflammatory factors such as IL-1*β* and IL-6 and plays a major role in the system's stress response. In our study, we observed an increase of proinflammatory molecules such as TNF-*α*, IL6, and IL-18 (Figures 1(a)–1(c)). IL-18 plays an important role in apoptosis and inflammatory diseases together with caspase-1. This proinflammatory response in our rat model suggests a successful establishment of lung injury on ischemia reperfusion. To further validate the establishment of lung injury, we looked at the histology of the lung tissue by hematoxylin and eosin staining. As demonstrated by other groups [24, 47], the histology sections of IR-induced rat lungs showed characteristic observations such as vascular congestion, thickening of alveolar septa, pulmonary edema, red blood cells, and inflammatory infiltrate that are specific to IR injury. All of the above variables were observed in our lung model, confirming lung tissue injury upon ischemia reperfusion (Figures 1(d) and 1(e)).

Recent findings have attributed a key role to miRNAs in the pathophysiology of ischemia reperfusion injury. miRNAs do so by altering the expression levels of genes involved in response to ischemia reperfusion [30]. Several important miRNAs have been studied in pulmonary disorders, ischemia reperfusion injury, and solid organ transplantation [30, 48, 49]. Unbiased approach to study miRNAs involved in ischemia reperfusion has been done in heart and renal tissues [26, 50] but not many high-throughput studies are done from the lung tissue, an organ known to be affected in most of the transplantation surgeries. Thus, we performed high-throughput analysis of miRNAs from rat lung model to establish the signature of miRNAs involved in ischemia reperfusion-mediated inflammatory response and subsequent injury.

Our high-throughput data shows that 69 miRNAs show significant deviation from the sham model. Of these, 43 miRNAs show significant upregulation and 26 miRNAs show significant downregulation as compared to the sham model (Figures 2(b) and 2(c)). As this analysis was done from the rat lung tissue, it reflected the miRNA expression status in the lung tissue in response to ischemia reperfusion-mediated signaling. From studies, we know that miRNAs can bring about translation repression of its target mRNAs; the differentially regulated miRNAs can lead to changes in the expression levels of several of target mRNAs [51–54]. It can be speculated that downregulation of certain miRNAs in the lung IRI would lead to translation upregulation of their target mRNAs and synthesis of new proteins required in immune and inflammatory response. On the other hand, upregulation of certain miRNAs would lead to inhibition of translation of several of the target mRNAs required for cell proliferation and cell survival [55–57]. Various miRNAs are known to be upregulated on acute lung injury such as miRNA-21 and miRNA-32-3p, whereas some are shown to be downregulated such as miR-155, let-7, and miR-146, all of them involved in inflammatory and TGF-*β* signaling miRNA gene networks. miRNA 127 that is downregulated on bleomycin-induced lung injury is known to target Fc receptor for IgG (IgG Fc*γ*RI-CD64), receptor part of clearance of immune complexes and proinflammatory cytokine release systems [49]. Another important study showed the role of miRNA-16 in hyperoxia leading to pulmonary edema (REF). On hyperoxia, levels of miRNA-16 dropped leading to increased expression of serotonin transporters [58]. Additionally, these miRNAs can be used as potential biomarkers in diagnosing inflammatory response in patients that have undergone transplantations. The detection of miRNAs can be used for timely interventions of medication in cases of rejection or organ failure.

Further, the validation of the top miRNAs was carried out by qPCR using specific primers for each of the selected miRNAs (Table 1). For the validation, both *in vivo* and *in vitro* systems were used. qPCR validation from the rat lung tissue sample which underwent ischemia reperfusion corroborated well with the high-throughput results (Figure 3(a)). The *in vitro* model was established with the rat type II alveolar cells that underwent hypoxia and reoxygenation, mimicking ischemia reperfusion in the lung tissue. qPCR for miRNAs from these cells also showed the same trend as that of the *in vivo* model (Figure 3(b)). Out of the six miRNAs that were validated by qPCR, rno-miR-18a-5p showed a consistent and significant downregulation upon IR or H/R. miRNA-18a-5p has been shown to be downregulated in the plasma of patients with acute heart failure and produced in cortical bone stem cells in response to heart injury [59, 60]. While there are no studies on the role of this miRNA in lung injury, our study for the first time provides a novel miRNA candidate that plays a role in lung ischemia reperfusion injury.

**Figure 9:**
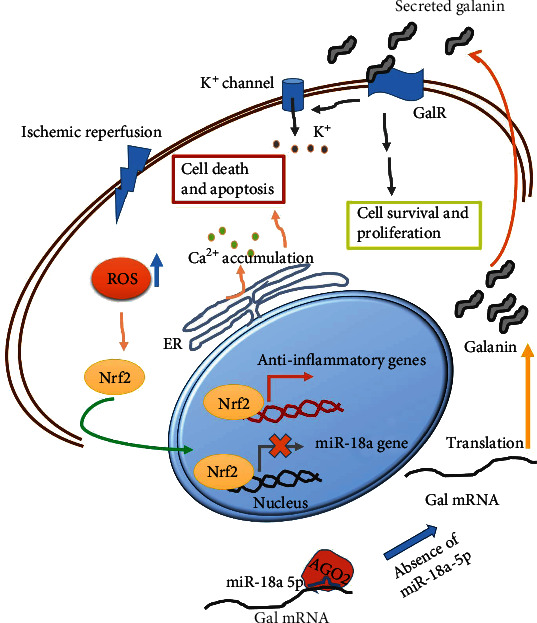
Model depicting the molecular pathways activated on ischemic reperfusion and the antioxidant protective mechanism via increased synthesis of Galanin.

The role of miR-18a-5p in mediating the response to IR was characterized. Firstly, the gene targeted by this miRNA was found by prediction and site mutagenesis analysis. Gal mRNA was found to be a valid target of miR-18a-5p and is upregulated on IR, both at the mRNA level (Figures 4(a) and 4(b)) and at the protein level (Figure 8(b)). Mutation in the miR-18a-5p binding site of the 3′UTR of the Gal mRNA does not affect the translation efficiency of the luciferase construct harboring this 3′UTR, providing evidence of the interaction with miR-18a-5p and leading to translation suppression. Gal is a 29-30 aa long neuropeptide, widely distributed in the central and peripheral nervous system [33]. Such an increase in Gal expression is also observed in neurons of the hippocampal hilar region that underwent IR. Gal is known to activate potassium channels, which hyperpolarize the neurons, inhibiting glutamate-mediated neurotoxicity [61]. While Gal binds to three G-protein-coupled receptor subtypes (GalR1, R2, and R3), its neuroprotective and proliferative effect is known to be carried downstream of GalR2 [62–64]. On IR in the rat lung tissue, due to the decrease in the levels of miR-18a-5p, the translation of Gal mRNA increases leading to increased protein production. Thus, it can be speculated that newly synthesized Gal in the lung tissue is secreted and activates the GalR2 receptor leading to the activation of pathways that promote cell survival and proliferation (Figure 9). GalR2 activation can in turn activate the potassium channels and lead to balancing of intracellular ions and leading to ATP stability and mitochondrial health [65].

In order to elucidate the molecular mechanism orchestrating downstream of ischemia reperfusion injury, we looked at various markers of cellular stress such as ROS generation, cell survival and apoptosis, expression of apoptotic markers such as Bcl-2 and BAX, and Ca^2+^ accumulation [66, 67]. We observed a reduction in cell viability on H/R treatment in AT2 cells (Figure 6(a)) which can then be attributed to apoptosis as shown in Figures 6(b) and 6(c). Intriguingly, the apoptotic effects of H/R can be accentuated by the overexpression of miR-18a-5p mimics or diminished by inhibiting miR-18a-5p by inhibitor (Figure 6). Thus, the grasp of miR-18a-5p levels on the cellular phenotype during H/R can be credited to the expression levels of Gal, with increased levels of Gal, showing cytoprotective effect and increasing cell proliferation. This cytoprotective effect is also seen via ROS levels, Ca^2+^ accumulation, and the levels of apoptotic proteins on treatment with H/R and in the presence of miR-18a-5p mimics and inhibitor (Figures 6 and 7). miR-18a-5p not only regulates the levels of ROS and Ca^2+^ in the cells but also the apoptotic response via proteins Bcl-2 and BAX. Thus, these results point towards the intrinsic proapoptotic pathway [68] that is activated on H/R and the levels of miR-18a-5p regulate this pathway through controlling the levels of Gal expression (Figure 8).

An exciting observation was that the localization of transcription factor, Nrf2, was also dependent on the levels of miR-18a-5p. Nrf2 is a master regulator of cytoprotective genes and is known to be held up in the cytoplasm by Kelch-like ECH- associating protein 1 (Keap1). The Nrf2-Keap complex is sensitive to the cellular ROS levels and on oxidative stress gets rapidly accumulated in the nucleus. It heterodimerizes with small protein Maf and binds to the ARE promoter region of the Nrf2 target genes [40]. Remarkably, Nrf2 is also known to inhibit the transcription of miRNAs such as miR1 and miR206 during tumorigenesis [69]. Hence, it can be speculated that during the initial stages of reperfusion, the increase in cellular ROS drives Nrf2 into the nucleus leading to the activation of anti-inflammatory genes but inhibition of the miR-18a gene (Figure 8). This leads to the buildup of Gal through increased translation, which then further activates cell survival and proliferative pathways through GalR2. Exactly how Gal leads to cytoprotection and recovery from IR injury would be the subject of future research. In conclusion, we have opened a new arena by profiling miRNA levels on a global scale from rat lung IR model bringing miRNAs and their regulation of translation in the spotlight, especially through the example of miR-18a-5p/Gal mRNA which can be important for further mechanistic studies and therapeutic interventions.

## 5. Conclusions

In this study, we have provided insights into the molecular mechanism of the response to lung ischemic reperfusion. To start with, we enumerate the microRNAs that are differentially expressed on rat lung ischemic reperfusion injury as compared to untreated rat lung tissue. Six of the differentially expressed miRNAs were validated in *in vivo* and *in vitro* models, out of which miR-18a-5p showed consistent downregulation in both models on IR. We discovered that miR-18a-5p targets the neuropeptide Galanin mRNA and suppresses its translation. But the levels of miR-18a-5p are reduced on IR which leads to the increase in Galanin mRNA translation and protein production. Additionally, miR-18a-5p regulates the cellular ROS levels which in turn affect the cytosolic Ca^2+^ levels and apoptosis. The localization of transcription factor Nrf2 into the nucleus is also affected by the levels of miR-18a-5p. Thus, by regulating the levels of miR-18a-5p, IR leads to activation of both proapoptotic pathways which gets balanced by cytoprotective pathways activated by Galanin neuropeptide, potentially leading to survival. This study highlights the role of miRNAs in response to lung ischemic reperfusion injury, not reported earlier in the field.

## Figures and Tables

**Table 1:** List of primers used for quantitative analysis of miRNAs.

Primer name	Base sequence (5′-3′)
U6 F	CTCGCTTCGGCAGCACA
U6 R	AACGCTTCACGAATTGTGCGT
rno-miR-667-5p RT	CTCAACTGGTGTCGTGGAGTCGGCAATTCAGTTGAGGTGCTCAC
rno-miR-667-5p F	ACACTCCAGCTGGGCGGTGCTGGTGGAGCAGT
rno-miR-329-5p RT	CTCAACTGGTGTCGTGGAGTCGGCAATTCAGTTGAGGAAACAGA
rno-miR-329-5p F	ACACTCCAGCTGGGAGAGGTTTTCTGGGTCTC
rno-miR-540-3p RT	CTCAACTGGTGTCGTGGAGTCGGCAATTCAGTTGAGGCCCAGGA
rno-miR-540-3p F	ACACTCCAGCTGGGAGGTCAGAGGTCGATC
rno-miR-335 RT	CTCAACTGGTGTCGTGGAGTCGGCAATTCAGTTGAGACATTTTT
rno-miR-335 F	ACACTCCAGCTGGGTCAAGAGCAATAACGAA
rno-miR-18a-5p RT	CTCAACTGGTGTCGTGGAGTCGGCAATTCAGTTGAGCTATCTGC
rno-miR-18a-5p F	ACACTCCAGCTGGGTAAGGTGCATCTAGTGC
rno-miR-20b-5p RT	CTCAACTGGTGTCGTGGAGTCGGCAATTCAGTTGAGCTACCTGC
rno-miR-20b-5p F	ACACTCCAGCTGGGCAAAGTGCTCATAGTGC
R universal primer	TGGTGTCGTGGAGTCG

**Table 2:** List of kits used for detection of ELISA.

Name	Brand	Item no.
Rat IL-6 ELISA kit	Dr. Biology	EK0412
Rat IL-18 ELISA kit	Dr. Biology	EK0592
Rat TNF-*α* ELISA kit	Dr. Biology	EK0526

**Table 3:** List of miRNAs selected for qPCR validation.

No.	Name	Sequence (5′-3′)	Up/down
1	rno-miR-667-5p	CGGUGCUGGUGGAGCAGUGAGCAC	Up
2	rno-miR-329-5p	AGAGGUUUUCUGGGUCUCUGUUUC	Up
3	rno-miR-540-3p	AGGUCAGAGGUCGAUCCUGGGC	Up
4	rno-miR-335	UCAAGAGCAAUAACGAAAAAUGU	Down
5	rno-miR-18a-5p	UAAGGUGCAUCUAGUGCAGAUAG	Down
6	rno-miR-20b-5p	CAAAGUGCUCAUAGUGCAGGUAG	Down

## Data Availability

The sequencing data obtained from this study has been uploaded on the Gene Expression Omnibus database (GEO) and the accession number is GSE161520.

## Supplementary Materials

Supplementary 1 Supplementary Table S1: list of differentially expressed miRNAs on IR lung injury from rat lungs.

Supplementary 2 Supplementary Table S2: list of genes targeted by miR-18a-5p.

Supplementary 3 Supplementary Figure 1: experimental flow of library preparation and sequencing analysis.

Supplementary 4 Supplementary Figure 2: prediction of miR-18a-5p binding on the 3′UTR of Gal mRNA.
