# Supplementary figures and images for: Novel Role of miR-18a-5p and Galanin in Rat Lung Ischemia Reperfusion-Mediated Response
Source: Oxid Med Cell Longev. 2021 Aug 14;2021:6621921. doi: 10.1155/2021/6621921 (PMC8420977; doi:10.1155/2021/6621921)

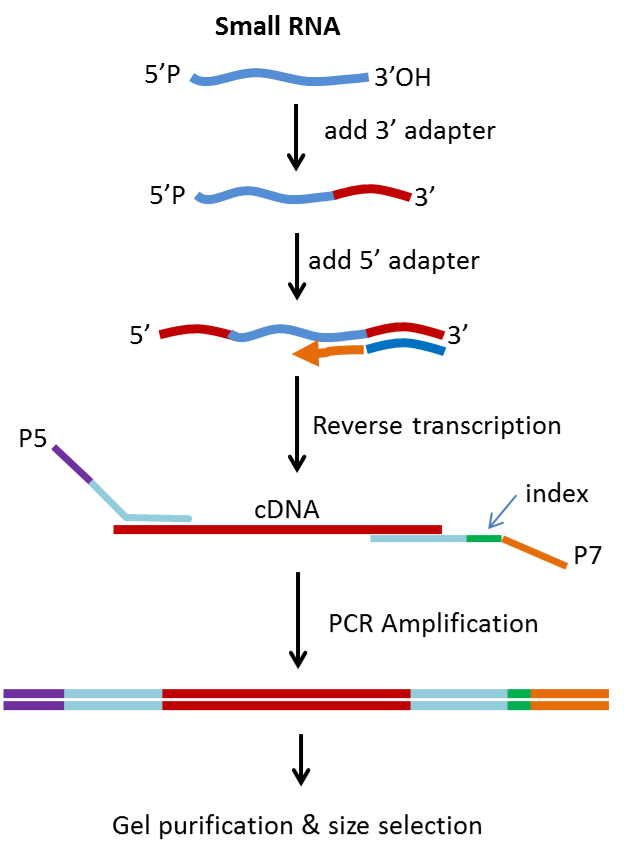

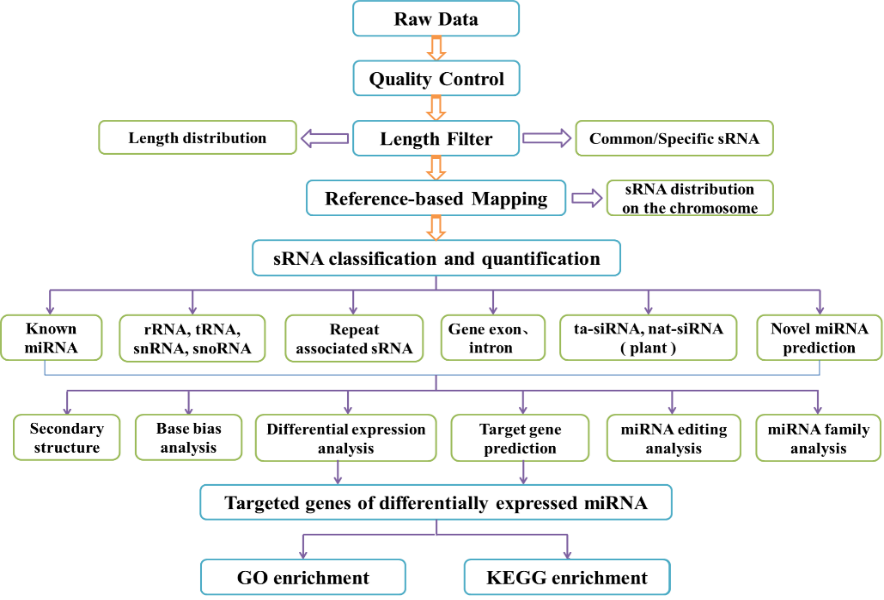


**A**

**B**

Supplement: Supplementary 3 — Supplementary Figure 1: experimental flow of library preparation and sequencing analysis. [file 6621921.f3.docx]

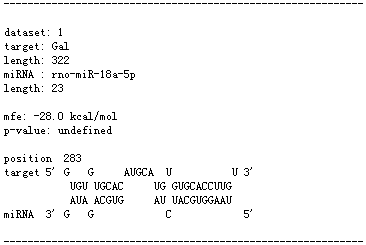

Supplement: Supplementary 4 — Supplementary Figure 2: prediction of miR-18a-5p binding on the 3′UTR of Gal mRNA. [file 6621921.f4.docx]
